# Do Symptoms and Serum Calcium Levels Affect the Results of Surgical Treatment of Primary Hyperparathyroidism?

**DOI:** 10.1155/2019/2150159

**Published:** 2019-07-01

**Authors:** QingAn Yu, KunPeng Liu, DaKun Ma, ChangMing Xie, YaoHua Wu, WenJie Dai, HongChi Jiang

**Affiliations:** Department of Thyroid Surgery, The First Affiliated Hospital Harbin Medical University, Harbin 150001, China

## Abstract

**Introduction:**

The purpose of this study was to investigate the difference in surgical outcomes between symptomatic and asymptomatic patients with primary hyperparathyroidism (PHPT) and between patients with high serum calcium and those with normal blood calcium, as well as to explore the epidemiological trend of PHPT in northern China.

**Methods:**

Clinicopathologic data of 197 patients (50 men and 147 women) with PHPT who underwent surgery at the First Affiliated Hospital of Harbin Medical University from 2008 to 2017 were analyzed. Changes in clinicopathology were compared among different subgroups of patients. Patients were categorized into subgroups based on serum calcium levels, whether or not they presented with symptoms, and admission time.

**Results:**

Of the total patients, 82.23% had hypercalcemic primary hyperparathyroidism (HCPHPT), 17.77% had normocalcemic primary hyperparathyroidism (NCPHPT), 45.18% had symptomatic primary hyperparathyroidism (SPHPT), and 54.82% had asymptomatic primary hyperparathyroidism (ASPHPT). Seventy-seven cases of PHPT involved thyroid nodules, with 22 confirmed as papillary thyroid carcinoma, and 29 confirmed as nodular goiter. There was no significant difference in the success rate of surgery, postoperative recurrence rate, and the symptoms of temporary hypocalcemia between the HCPHPT and NCPHPT groups, and between the SPHPT and ASPHPT groups. The incidence of PHPT has increased threefold since 2013.

**Conclusions:**

Symptoms and serum calcium levels did not affect the results of surgical treatment for PHPT. The incidence of PHPT in northern China is increasing. Moreover, PHPT manifestation has shifted from the symptomatic to the asymptomatic form. Thyroid surgery should be performed in PHPT patients with thyroid nodules.

## 1. Introduction

Before the 1970s, primary hyperparathyroidism (PHPT) was considered a rare disease, and patients presented with marked hypercalcemia and bone lesions or osteitis fibrosa cystica. However, with the advent of the multifunction autoanalyzer in the 1970s, the clinical phenotype changed. PHPT has become the third major endocrine disease in western countries following diabetes and osteoporosis [1]. According to reports from western countries, the incidence of PHPT has undergone 2 peaks. The first peak occurred during 1974-1982 and was mainly due to the increased prevalence of serum calcium screenings. The second peak occurred during 2000-2010 and was probably due to the targeting of PHPT screening for osteoporosis patients [2–4]. Not only the incidence but also the form of PHPT is changing. PHPT is changing mainly from hypercalcemic primary hyperparathyroidism (HCPHPT) to normocalcemic primary hyperparathyroidism (NCPHPT) [5, 6] and from symptomatic hyperparathyroidism (SPHPT) to asymptomatic primary hyperparathyroidism (ASPHPT) [7, 8].

It is unknown whether the changes in the forms of PHPT will affect the therapeutic outcomes of PHPT. In this study, we reviewed the treatment of PHPT over the past 10 years, discussed the abovementioned problem, and analyzed the incidence and clinical characteristics of PHPT in northern China.

## 2. Materials and Methods

### 2.1. Study Participants

Study participants were selected from patients treated at the Department of General Surgery at the First Affiliated Hospital of Harbin Medical University from 2008 to 2017. Patients who met the diagnostic criteria for PHPT, underwent parathyroidectomy, and had pathologically confirmed parathyroidism were included in this study. This study was approved by the ethics committee of the First Affiliated Hospital of Harbin Medical University. Demographic and clinicopathological information was collected regarding patients' sex, age, pre-operative biochemical examination, postoperative biochemical examination, pathological findings, operative cure rate, postoperative recurrence rate, and other clinical characteristics.

HCPHPT is characterized by high serum calcium levels and elevated parathyroid hormone (PTH) [9]. The diagnostic criteria for HCPHPT were as follows: histologically proven PHPT diagnosis, hypercalcemia (calcium level >2.6 mmol/L) with elevated serum PTH, and hypercalcemia with no specified cause (e.g., thiazide diuretics, cancer, creatinine level >176.8 mmol/L, or lithium therapy). NCPHPT is characterized by normal serum calcium levels and continuously elevated PTH [10]. The diagnostic criteria for NCPHPT were as follows: histologically proven PHPT diagnosis; normal serum calcium (calcium level ≤2.6 mmol/L) and high PTH levels; serum 25-hydroxyvitamin D (25[OH]D) levels above 30 ng/mL; the absence of bisphosphonates, thiazide diuretics, anticonvulsants, or lithium use; glomerular filtration rate greater than 60 mL/min; and the absence of other metabolic bone diseases or gastrointestinal diseases associated with malabsorption or liver disease. SPHPT is characterized by abnormal elevated PTH levels and is associated with organ-specific symptoms, including renal disease, bone disease, gastrointestinal symptoms, mental symptoms, neuromuscular symptoms, cognitive dysfunction, and cardiovascular system symptoms [11–18]. ASPHPT is characterized by elevated PTH levels in the presence or absence of hypercalcemia with no obvious clinical symptoms or target organ manifestations [19].

### 2.2. Evaluations and Biochemical Analysis

Patients underwent standard evaluations that included personal histories, family histories, physical examinations, auxiliary examinations, and blood and urinary biochemical tests. Total serum calcium (reference range, 2.08–2.6 mmol/L), phosphate (0.96–1.62 mmol/L), creatinine (35–80 *μ*mol/L), albumin (34–54 g/L), and total serum alkaline phosphatase (AKP) (reference range, 40–150 IU/L) levels were measured using an autoanalyzer (Beckman Coulter, Fullerton, California). Serum calcium was corrected using the following formula: corrected calcium (mg/dL) = (0.8 [4.0 - patient's albumin (g/dL)]) + total calcium (mg/dL). PTH level was measured using an electrochemiluminescence immunoassay (ECLIA) (reference range, 10–69 pg/mL) on a fully automated analyzer E 601 (Roche Diagnostics GmbH, Mannheim, Germany). The serum 25(OH)D concentration was measured using an enzyme immunoassay (assay reference range, 17.88–57.6 ng/mL [44.7–144 nmol/L]; Immunodiagnostic Systems, Boldon, United Kingdom).

All patients underwent cervical ultrasonography and/or technetium-99m-sestamibi parathyroid scintigraphy. All patients underwent urinary ultrasonography and/or abdominal X-ray radiography. Bone mineral density (BMD) was determined using a dual-energy X-ray absorptiometer (Lunar Prodigy; GE Medical Systems, Madison, Wisconsin).

### 2.3. Study Groups

This study analyzed data from 3 sets of study groups. Based on their serum calcium levels, PHPT patients were divided into either an HCPHPT group or an NCPHPT group. PHPT patients were also divided into either an SPHPT group or an ASPHPT group based on whether they presented with symptoms or not. The clinicopathologic features and surgical results of each group were compared. Finally, according to the time of admission, patients were divided into group A for those admitted in the period 2008-2012 and group B for those admitted in the period 2013-2017. The trend of PHPT in northern China was examined by comparing groups A and B.

In addition, symptoms in each group were further divided into type I and type II symptoms. Type I symptoms were classified as single or multiple symptoms, while type II symptoms were classified according to specific symptom types.

### 2.4. Statistical Analysis

The data were analyzed using IBM SPSS software (Version 19.0, IBM Corp., Armonk, NY). Continuous variables were expressed as means and standard deviations. For data that were normally distributed and had homogeneous variances, the t-test was used to assess differences between groups. For data that were not normally distributed or did not have homogeneous variances, the Mann-Whitney U rank sum test was used for analysis. Categorical variables and nonparametric variables were examined by chi-square test or Fisher's exact test. P-values <0.05 were considered statistically significant.

## 3. Results

### 3.1. Overall Clinicopathologic Data

**Figure 1:**
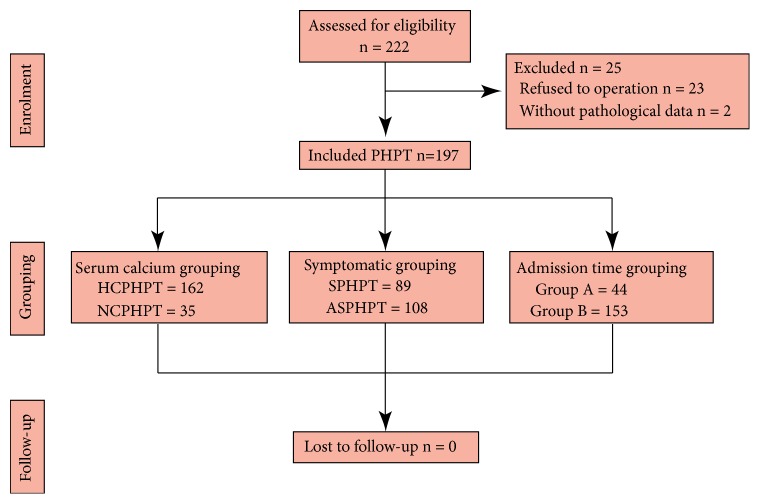
Study flow chart. Abbreviations: PHPT, primary hyperparathyroidism; HCPHPT, hypercalcemic primary hyperparathyroidism; NCPHPT, normocalcemic primary hyperparathyroidism; SPHPT, symptomatic primary hyperparathyroidism; ASPHPT, asymptomatic primary hyperparathyroidism; Group A: Patients who have been receiving treatment in 2008-2012; Group B: Patients who have been receiving treatment in 2013-2017.

A total of 222 cases of PHPT were included, of which 23 cases were excluded because of refusal to undergo surgery, and 2 cases were excluded due to radiofrequency ablation without pathological data (Figure 1). Finally, 197 cases (50 men and 147 women) were included (Figure 1). The average age was 49 years (interquartile range: 43-57) (Table 1). Of the total patients, 162 (82.23%) presented with HCPHPT, and 89 (45.18%) presented with SPHPT. In addition, 161 (81.73%) patients had parathyroid adenoma, 30 (15.23%) had hyperplasia, 2 (1.02%) had carcinoma, and 4 (2.03%) had adenoma concurrent with hyperplasia. Among the patients with parathyroid adenoma, single adenoma accounted for 96.27% of cases, and among the patients with parathyroid hyperplasia, single parathyroid hyperplasia accounted for 66.67% of cases. Seventy-seven (39.09%) patients had concurrent thyroid nodules, of which 51 patients underwent thyroidectomy and parathyroidectomy simultaneously. Of the 51 patients with simultaneous thyroidectomy, 22 had papillary thyroid carcinoma, and 29 had a nodular goiter. The cure rate of parathyroidectomies was 96.45%. Seven cases were unsuccessful, with 4 cases having ectopic parathyroid adenoma; 1 case had multiple parathyroid adenomas that were not removed, and 2 cases had multiple parathyroid hyperplasia which was not completely removed. After surgery, 3 cases (1.52%) had a relapse.

### 3.2. Comparison between the HCPHPT and NCPHPT Groups

The HCPHPT group contained 162 (82.23%) cases, and the NCPHPT group contained 35 (17.77%) cases. There was no significant difference in age and sex between the 2 groups (P >0.05, Table 2). In the preoperative biochemical examination, the concentrations of PTH and serum calcium in the HCPHPT group were significantly higher than those in the NCPHPT group (P <0.0001), but there was no significant difference in AKP and serum phosphorus between the 2 groups (P >0.05). Patients in the HCPHPT group were more likely to present with symptoms than patients in the NCPHPT group (P <0.05, Table 2); however, there was no significant difference in type I or II symptoms and composition between the 2 groups (P >0.05). Both postoperative serum calcium and phosphorus in the HCPHPT group were significantly higher than those in the NCPHPT group (P <0.05, Table 3). There was no significant difference between the 2 groups regarding the concentration of postoperative PTH or the diameter, type, and number of lesions. Moreover, the success rate of the operation, the postoperative recurrence rate, and the symptoms of temporary hypocalcemia were not significantly different between the groups (Table 3).

### 3.3. Comparison between the SPHPT and ASPHPT Groups

The SPHPT group contained 89 (45.18%) cases, and the ASPHPT group contained 108 (54.82%) cases. There was no significant difference in age and sex between the 2 groups (P >0.05, Table 4). In the preoperative biochemical examination, the concentrations of PTH, AKP, and calcium in the SPHPT group were significantly higher than those in the ASPHPT group (P <0.0001), but there was no significant difference in the serum phosphorus levels between the 2 groups (P >0.05). The postoperative serum calcium and lesion diameter of the SPHPT group were larger than those in the ASPHPT group, and the concentrations of postoperative PTH and serum phosphorus were lower than those in the ASPHPT group (P <0.05) (Table 5). Concerning the pathological type of the lesion, the proportion of adenoma in the SPHPT group was significantly higher than that in the ASPHPT group, and the proportion of hyperplasia was significantly lower than that in the ASPHPT group (P <0.05). There was no significant difference in the number of lesions between the groups. The number of patients that underwent simultaneous thyroidectomy was significantly different between the SPHPT group (11.24%) and the ASPHPT group (37.96%) (P <0.0001). Operative success rate, postoperative recurrence rate, and temporary symptoms of hypocalcemia were not significantly different between the groups (Table 5).

### 3.4. Incidence of PHPT between 2008-2012 and 2013-2018

The number of patients with PHPT was significantly higher in group B (the last 5 years) than in group A (the first 5 years), and there was no significant difference in sex, age, and pre-operative calcium between the groups (Table 6). The concentrations of pre-operative PTH and AKP in group B were significantly lower than those in group A (P <0.05), while that of pre-operative serum phosphorus in group B was significantly higher than that in group A (P = 0.003). In group A, 84.09% (n = 37) of patients showed clinical symptoms which was a significantly higher proportion than that of patients with clinical symptoms in group B (33.99%, n = 52) (P <0.0001). The proportion of patients with multiple symptoms was significantly higher in group A than in group B (P=0.007), and the prevalence of renal and skeletal diseases was significantly higher in group A than in group B (P<0.001).

## 4. Discussion

This study examined whether there were differences between the surgical outcomes of patients with SPHPT versus ASPHPT and HCPHPT versus NCPHPT. By comparing the clinical features and surgical outcomes of the groups, we found no difference between the outcomes of surgical treatment. Moreover, the analysis of clinical features showed that PHPT combined with thyroid nodules was more common. By comparing the treatment of hyperparathyroidism during the last 10 years, the number of PHPT patients in the last 5 years was found to be significantly higher than that in the preceding 5 years, which was consistent with the overall increasing trend in the incidence of PHPT in recent years.

Our data showed that HCPHPT is the most common form of PHPT in northern China, which is consistent with other research centers in China [20]. The pathological mechanism of NCPHPT is unclear; however, there are 2 main current hypotheses. The first hypothesis is that NCPHPT is an early-stage or attenuated PHPT [21]. Another hypothesis is that some of the tissues of patients with NCPHPT may be resistant to PTH [22]. Compared with patients with normal blood calcium, the surgical outcome of patients with elevated blood calcium was not significantly different. However, the incidence of clinical symptoms was higher in patients with NCPHPT than in patients with HCPHPT, but there was no statistical difference in the composition of the symptoms. Although the timing of surgical treatment for NCPHPT patients is somewhat controversial, studies have shown that surgery is beneficial in preventing a decrease in bone density [23].

In this study, the proportion of SPHPT patients was comparable to that of ASPHPT patients, but it was significantly lower than that reported in developed countries (70%–81.8%) [24–26]. Preoperative PTH, AKP, and calcium of the SPHPT patients were significantly higher than those of the asymptomatic group. In addition, patients with clinical symptoms were more likely to be diagnosed and have greater opportunities for surgical treatment. In terms of surgical outcomes, there was no statistical difference between symptomatic and asymptomatic patients. However, surgery can significantly improve the postoperative quality of life of patients, especially regarding neurocognition, sleep, and blood pressure [27] and also improve bone fiber results and increase bone strength [28]. Therefore, surgery was relatively large for patients with PHPT, especially those with SPHPT.

In this study, 39.09% of PHPT patients had concurrent thyroid nodules. PHPT combined with thyroid nodules has attracted much attention in recent years, especially when combined with non-medullary thyroid carcinoma. The incidence of PHPT combined with thyroid nodules has increased threefold [29, 30]. Neslihan et al. [31] reported that 66.7% of PHPT patients also had thyroid nodules; the postoperative pathological results showed that 79.2% of the thyroid nodules were benign thyroid diseases, and 20.8% were papillary thyroid carcinoma. Recent studies show that a total of 60% of patients with PHPT have thyroid disease, while almost one-third of patients are not aware of this disease before the diagnosis of PHPT [32]. The incidence of PHPT coupled with either thyroid nodules or thyroid cancer is increasing, which makes diagnosis and treatment more complex. Among the 51 cases with thyroid nodules complicated with PHPT, 43.14% were combined with papillary thyroid carcinoma. In the past, the proportion of PHPT cases with thyroid cancer was 10.87%–12.9% [33, 34]. Therefore, it is necessary to perform surgical treatment for PHPT combined with thyroid disease when the pathological hyperthyroidism is removed by surgery.

In this study, the proportion of female patients was significantly higher than that of male patients, accounting for 74.62% of the total patients. This difference has been observed in multiple regions and populations. In a study of 464 PHPT patients in India, women accounted for 70.47% of all patients [35], and in a single-center study of 300 PHPT patients in Rhode Island Hospital, women accounted for 74% of all patients [36]. PHPT is more common in women than in men, with a 3:1 sex ratio [37]. According to an epidemiological survey using big data, the prevalence of PHPT in the United States has been estimated at 23 cases per 10,000 women and 8.5 cases per 10,000 men, with an incidence of 65.5 cases per 100,000 person-years in women and 24.7 cases per 100,000 person-years in men [38]. The specific reasons for these differences remain unexplained. In this study, the average age of PHPT patients was lower than that reported in previous studies [36, 38], especially in developed regions; however, it was higher than the average age (41 ± 14 years) reported in the previous Indian study [35], which may be related to the regional economy and healthcare of residents as well as other factors.

The limitations of this study are as follows. First, this was a single-center retrospective study with bias in patient selection. Second, there was insufficient power to reliably evaluate the difference in cure rate between the normocalcemic and hypercalcemic groups. However, considering the proportion of patients in the present study and assuming the power was 0.9, 737 patients would have to be included in the HCPHPT group and 160 in the NCPHPT group to make the difference in the cure rate of 6.1% statistically significant.

## 5. Conclusion

Parathyroidectomy is a safe and effective treatment for HCPHPT and NCPHPT. Although patients with SPHPT are different from those with ASPHPT regarding some indicators, the differences do not affect the results of surgical treatment. We also found that the incidence of PHPT in northern China is increasing. Thyroid surgery should be performed for PHPT patients with thyroid nodules. In future studies, the hypercalcemic group could be further divided into subgroups according to the critical blood calcium value of 3.49 mmol/L to compare differences in surgical treatment outcome between PHPT patients with normal blood calcium, hypercalcemia, and severe hypercalcemia.

## Figures and Tables

**Table 1:** Clinicopathological data of all patients.

Index	Patients (n=197)
Age, y (IQR)	49 (43-57)
Female	147 (74.62%)
Male	50 (25.38%)
HCPHPT	82.23% (162/197)
NCPHPT	17.77% (35/197)
SPHPT	45.18% (89/197)
ASPHPT	54.82% (108/197)
Parathyroid adenoma	81.73% (161/197)
Parathyroid hyperplasia	15.23% (30/197)
Parathyroid carcinoma	1.02% (2/197)
Parathyroid adenoma with hyperplasia	2.03% (4/197)
PHPT with thyroid nodules	39.09% (77/197)
Papillary thyroid carcinoma	43.14% (22/51)
Nodular goiter	56.86% (29/51)
Cure rate of PHPT	96.45% (190/197)
Recurrence rate of PHPT	1.52% (3/197)

Abbreviations: IQR, interquartile range; HCPHPT, hypercalcemic primary hyperparathyroidism; NCPHPT, normocalcemic primary hyperparathyroidism; SPHPT, symptomatic primary hyperparathyroidism; ASPHPT, asymptomatic primary hyperparathyroidism; PHPT, primary hyperparathyroidism.

**Table 2:** Comparison of preoperative data between HCPHPT group and NCPHPT group.

Index	HCPHPT group n=162	NCPHPT group n=35	*P *value
Age (y) ± SD	48.39±12.04	50.54±13.94	0.352
Sex			0.632
male	40 (24.69%)	10 (28.57%)	
female	122 (75.31%)	25 (71.43%)	
Preoperative PTH (uIU/ml) ± SD	781.65 ± 799.25	347.38 ± 372.56	<0.0001
Preoperative AKP (U/L) ± SD	295.37 ±365.35	174.99 ± 269.13	0.067
Preoperative Ca (mmol/L) ± SD	3.06 ± 0.38	2.37 ± 0.35	<0.0001
Preoperative P (mmol/L) ± SD	2.64 ± 15.25	0.93 ± 0.21	0.507
Patients with symptom			0.029
yes	48.77% (79/162)	28.57% (10/35)	
no	51.23% (83/162)	71.43% (25/35)	
Symptom type I			0.179
Single symptoms	54.43%(43/79)	80.00%(8/10)	
Multiple symptoms	45.57%(36/79)	20.00%(2/10)	
Symptom type II			0.540
nephrolithiasis	24.69% (40/162)	8.57% (3/35)	0.023
osseous lesion	24.07% (39/162)	17.14% (6/35)	0.506
gastrointestinal symptoms	8.64% (14/162)	5.71% (2/35)	0.742
others	15.43% (25/162)	2.86% (1/35)	0.053

Abbreviations: HCPHPT, hypercalcemic primary hyperparathyroidism; NCPHPT, normocalcemic primary hyperparathyroidism; PTH, parathyroid hormone; AKP, alkaline phosphatase.

**Table 3:** Comparison of postoperative data between HCPHPT group and NCPHPT group.

Index	HCPHPT group n=162	NCPHPT group n=35	*P *value
Postoperative PTH (uIU/ml) ± SD	50.29 ± 160.25	44.76 ± 40.58	0.840
Postoperative Ca (mmol/L) ± SD	2.39 ± 0.33	2.19 ± 0.18	<0.0001
Postoperative P (mmol/L) ± SD	0.86 ± 0.27	1.17 ± 0.25	<0.0001
Lesion diameter (cm)^a^	2.29 ± 1.23	1.97 ± 0.82	0.266
Pathological type			0.512
Parathyroid adenoma	82.72% (134/162)	77.14% (27/35)	
Parathyroid hyperplasia	13.58% (22/162)	22.86% (8/35)	
Parathyroid carcinoma	1.23% (2/162)	0.00% (0/35)	
Parathyroid adenoma with hyperplasia	2.47% (4/162)	0.00% (0/35)	
Cure rate of PHPT	97.53% (158/162)	91.43% (32/35)	0.108
Recurrence rate of PHPT	1.85% (3/162)	0.00% (0/35)	> 0.99
Incidence of temporary hypocalcemia	13.58% (22/162)	20.00% (7/35)	0.428

a. For multiple lesion tissues, the diameter of the largest lesion tissue was calculated.

Abbreviations: HCPHPT, hypercalcemic primary hyperparathyroidism; NCPHPT, normocalcemic primary hyperparathyroidism; PTH, parathyroid hormone; PHPT, primary hyperparathyroidism.

**Table 4:** Comparison of preoperative data between SPHPT group and ASPHPT group.

Index	SPHPT group n=89	ASPHPT group n=108	*P *value
Age (y) ± SD	47.93 ± 12.29	49.54 ± 12.33	0.394
Sex			0.262
male	26 (29.21%)	24 (22.22%)	
female	63 (70.79%)	84 (77.78%)	
Preoperative PTH (uIU/ml) ± SD	1110.69 ± 1058.09	435.98 ± 396.14	< 0.0001
Preoperative AKP (U/L) ± SD	353.67 ± 418.62	190.25 ± 201.31	< 0.0001
Preoperative Ca (mmol/L) ± SD	3.12 ± 0.51	2.80 ± 0.38	< 0.0001
Preoperative P (mmol/L) ± SD	0.94 ± 0.42	0.96 ± 0.32	0.702
Symptom type I			
Single symptoms	57.30%(51/89)	-	
Multiple symptoms	42.70%(38/89)	-	
Symptom type II			
nephrolithiasis	48.31%(43/89)	-	
osseous lesion	50.56%(45/89)	-	
gastrointestinal symptoms	17.98%(16/89)	-	
others	29.21%(26/89)	-	

Abbreviations: SPHPT, symptomatic primary hyperparathyroidism; ASPHPT, asymptomatic primary hyperparathyroidism; PTH, parathyroid hormone; AKP, alkaline phosphatase.

**Table 5:** Comparison of postoperative data between SPHPT group and ASPHPT group.

Index	SPHPT group n=89	ASPHPT group n=108	*P *value
Postoperative PTH(uIU/ml) ± SD	31.94 ± 47.55	63.48 ± 192.00	0.031
Postoperative Ca(mmol/L) ± SD	2.42 ± 0.39	2.31 ± 0.23	< 0.0001
Postoperative P(mmol/L) ± SD	0.82 ± 0.26	0.99 ± 0.30	< 0.0001
Lesion diameter(cm)^a^	2.43 ± 1.17	2.07 ± 1.22	0.034
Pathological type			0.053
Parathyroid adenoma	88.76% (79/89)	75.93% (82/108)	0.026
Parathyroid hyperplasia	8.99% (8/89)	20.37% (22/108)	0.029
Parathyroid carcinoma	0.00% (0/89)	1.85% (2/108)	0.502
Parathyroid adenoma with hyperplasia	2.25% (2/89)	1.85% (2/108)	> 0.99
Combined thyroidectomy			< 0.0001
yes	11.24% (10/89)	37.96% (41/108)	
no	88.76% (79/89)	62.04% (67/108)	
Cure rate of PHPT	96.63% (86/89)	96.30%(104/108)	> 0.99
Recurrence rate of PHPT	1.12% (1/89)	1.85% (2/108)	> 0.99
Incidence of temporary hypocalcemia	13.48% (12/89)	15.74% (17/108)	0.691

a. For multiple lesion tissues, the diameter of the largest lesion tissue was calculated.

Abbreviations: SPHPT, symptomatic primary hyperparathyroidism; ASPHPT, asymptomatic primary hyperparathyroidism; PTH, parathyroid hormone; PHPT, primary hyperparathyroidism.

**Table 6:** Clinical data of group A and group B.

Index	Group A n=44	Group B n=153	*P *value
PHPT	22.34% (44/197)	77.66% (153/197)	
Age (y) ± SD	46.00 ± 12.30	49.43 ± 12.35	0.106
Sex			0.948
male	25.00% (11/44)	25.49% (39/153)	
female	75.00% (33/44)	74.51% (114/153)	
Preoperative PTH	1025.54 ± 887.69	657.10 ± 806.75	0.01
Preoperative AKP	456.45 ± 551.29	221.70 ± 248.84	0.008
Preoperative Ca	3.01 ± 0.40	2.93 ± 0.48	0.297
Preoperative P	0.80 ± 0.24	0.95 ± 0.38	0.003
SPHPT	84.09% (37/44)	33.99% (52/153)	< 0.0001
Symptom type I			0.007
Single symptoms	40.54%(15/37)	69.23%(36/52)	
Multiple symptoms	59.46%(22/37)	30.77%(16/52)	
Symptom type II			
nephrolithiasis	56.82%(25/44)	11.76%(18/153)	< 0.0001
osseous lesion	52.27%(23/44)	14.38%(22/153)	< 0.0001
gastrointestinal symptoms	9.09%(4/44)	7.84%(12/153)	0.759
others	18.18%(8/44)	11.76%(18/153)	0.268

Group A: Patients who have been treated for 2008-2012; Group B: Patients who have been treated for 2013-2017.

Abbreviations: PHPT, primary hyperparathyroidism; PTH, parathyroid hormone; AKP, alkaline phosphatase.

## Data Availability

The data used to support the findings of this study are available from the corresponding author upon request.
